# A Two-Layer SVM Ensemble-Classifier to Predict Interface Residue Pairs of Protein Trimers

**DOI:** 10.3390/molecules25194353

**Published:** 2020-09-23

**Authors:** Yanfen Lyu, Xinqi Gong

**Affiliations:** 1 Mathematical Intelligence Application Lab, Institute for Mathematical Sciences, School of Math, Renmin University of China, Beijing 100872, China; lyf20130327wd@163.com; 2 Beijing Advanced Innovation Centre for Structural Biology, Tsinghua University, Beijing 100084, China

**Keywords:** a two-layer SVM ensemble-classifier, trimer protein–protein complexes, feature combination engineering

## Abstract

Study of interface residue pairs is important for understanding the interactions between monomers inside a trimer protein–protein complex. We developed a two-layer support vector machine (SVM) ensemble-classifier that considers physicochemical and geometric properties of amino acids and the influence of surrounding amino acids. Different descriptors and different combinations may give different prediction results. We propose feature combination engineering based on correlation coefficients and F-values. The accuracy of our method is 65.38% in independent test set, indicating biological significance. Our predictions are consistent with the experimental results. It shows the effectiveness and reliability of our method to predict interface residue pairs of protein trimers.

## 1. Introduction

Many protein complexes are formed by the interactions of multiple protein monomers. These complexes can carry out many biological functions, such as gene expression and regulation, signal transduction, or enzyme catalytic mechanisms [1]. Understanding the mechanisms of protein–polymer interactions can provide useful information for the design of protein polymer structures, protein functional annotation, and drug design [2]. The accurate prediction of interface residue pairs in polymeric proteins is an important part in the study of protein polymer interactions. Various experimental methods have been used in the research of protein polymers, such as X-ray crystallography and nucleic magnetic resonance. It is impossible and unrealistic to find the interface residue pairs for all protein polymers by an experimental method. Therefore, the prediction of protein polymer interface residue pairs has become an important question in bioinformatics.

An increasing number of computational methods have been developed to predict protein monomer binding sites and protein–protein interface residue pairs. In relation to this, Segura et al. [3] used a two-step Random Forest classifier to predict protein binding sites. Hwang et al. [4] proposed an index of Residue Contact Frequency to predict the protein monomer binding sites. Lyu et al. [5] defined an index of contact frequency from the protein–protein docking result to accurately predict protein–protein interface residue pairs. Ovchinnikov predicted residue–residue interactions across protein interfaces using evolutionary information [6]. There are many other methods that are not described here [7,8,9,10,11,12,13,14,15].

A higher number of protein monomers implies more complex interaction mechanisms of the protein polymer. At present, there are a few methods to predict interface residue pairs of protein trimer. Zhao et al. [16] took the sequence feature as input in multilayered Long Short-Term Memory networks to predict interface residue pairs of protein trimer. In this paper, we want to develop a new and effective method for prediction of interface residue pairs in protein trimers.

Properties of protein sequences depend on the composition and distribution of amino acids. The position and type of each amino acid in a protein sequence are unique, containing important structural and functional information. Therefore, for a specific position amino acid in the protein sequence, ideal descriptors should not only reflect the amino acid type but also structural information or the role played in the performance of the protein function. Based on previous studies on protein monomer binding sites and protein–protein interface residue pairs, we found some common properties that can allow to distinguish interface residues from the rest of the protein, using information of hydrophobicity, polarizability, solvent accessibility, and so on. We summarized these properties and divided them into two categories. The first category corresponded to amino acid physicochemical properties, including hydrophobicity [17,18,19], polarizability [20], and polarity [21]. The second category corresponded to residue geometric properties, such as accessible surface area (ASA) [22] and relative accessible surface area (RASA) [23].

In this paper, we defined an amino acid k-interval product factor to describe the influence of surrounding amino acids based on their physicochemical properties. Hence, we described a residue pair with three types of characteristics: amino acid physicochemical features, residue geometric features, and amino acid k-interval product factor. Different descriptors and different combinations may give different prediction results. We performed feature combination engineering based on correlation coefficients and F-values for all characteristics. In general, when the number of positive and negative samples in the dataset is seriously unbalanced, the accuracy and robustness of ensemble classifiers is higher than those of a single classifier (negative samples: noninterface residue pairs and positive samples: interface residue pairs). We trained a two-layer support vector machine (SVM) ensemble-classifier method to predict the interface residue pairs of protein trimers and tested it using an independent testing set. We also use different indicators to evaluate testing set results, which proves that our method is feasible.

**Figure 1:**
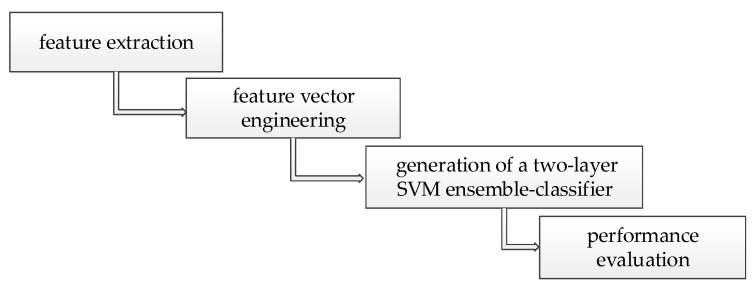
Key steps of the method.

In summary, our method was divided into four parts: feature extraction, feature vector engineering, generation of a two-layer SVM ensemble-classifier, and performance evaluation, as shown in Figure 1.

## 2. Materials and Methods

### 2.1. Dataset

In this paper, the dataset was collected from the Protein Data Bank based on following four requirements: the number of chains is 3, the length of each chain is between 20 and 500, it is obtained by X-ray experiment, and there are physical bindings between each two chains in one protein trimer. Two chains are defined as interactors if there are interface residue pairs between the two chains. (If the contact area between any two atoms from two residues is bigger than zero, we called these two residues in contact and these two residues are called an interface residue pair. Here, we used the Qcontacts software to calculate contact area between two atoms.) By this way, we collect 78 protein trimers (The data can be downloaded from Supplementary Materials). We randomly divided 78 protein trimers into training set and testing set, of which the number of training set is 52, accounting for 2/3 of the total, and the remaining 26 protein trimers are used as testing set (see Table 1).

### 2.2. Features Extraction

#### 2.2.1. The Amino Acid Physicochemical Features

A given protein sequence of length L defined on the base set Ω = {A, C, D, E, F, G, H, I, K, L, M, N, P, Q, R, S, T, V, W, Y} is expressed by

P = P_1_P_2_P_3_…P_L_. (1)

Different amino acids in Formula (1) have different physicochemical properties. These physicochemical properties, including hydrophobicity [17,18,19] and polarizability [20], play important roles in multiple protein monomer interactions. In this paper, we considered five physicochemical properties of amino acids: hydrophobicity [17,18,19], polarizability [20], polarity [21], secondary structure, and codon diversity [24]. Three versions of hydrophobicity were proposed by Tanford Charles, Jack Kyte, and David Eisenberg in 1962, 1982, and 1984, respectively. Other previous works, [24,25] have used the hydrophobicity value of Tanford [17] as a feature to identify protein–protein interactions. Some other authors [15,26] used the last two versions of the hydrophobicity value [18,19] as features to predict residue pairs in a protein–protein interface. Their prediction results were good, so we took three versions of the hydrophobicity index in our method. Appendix A Table A1 shows the numerical values of the five physicochemical properties for the 20 amino acids.

According to the corresponding properties of each amino acid, the protein sequence P can be converted into seven different number sequences (see Formula (2)). We used Φ^1^, Φ^2^, Φ^3^, Φ^4^, Φ^5^, Φ^6^, and Φ^7^ to represent the seven numerical sequences. These seven numerical sequences are the hydrophobicity number sequence 1, polarizability number sequence, polarity number sequence, secondary structure number sequence, codon diversity number sequence, hydrophobicity number sequence 2, and hydrophobicity number sequence 3.

(2) $$P\;=\;\{\begin{array}{c} \Phi_{1}^{1}\Phi_{2}^{1}\cdots \Phi_{L}^{1}\\ \Phi_{1}^{2}\Phi_{2}^{2}\cdots \Phi_{L}^{2}\\ \Phi_{1}^{3}\Phi_{2}^{3}\cdots \Phi_{L}^{3}\\ \Phi_{1}^{4}\Phi_{2}^{4}\cdots \Phi_{L}^{4}\\ \Phi_{1}^{5}\Phi_{2}^{5}\cdots \Phi_{L}^{5}\\ \Phi_{1}^{6}\Phi_{2}^{6}\cdots \Phi_{L}^{6}\\ \Phi_{1}^{7}\Phi_{2}^{7}\cdots \Phi_{L}^{7} \end{array}$$

where $\Phi_{1}^{1}$ is the hydrophobicity value of P_1_ in formula 1,${\;\Phi }_{2}^{1}$ is the hydrophobicity value of P_2_ in formula 1, and so on. $\Phi_{1}^{2}$ is the polarizability value of P_1_ in formula 1,${\;\Phi }_{2}^{1}$ is the polarizability value of P_2_ in formula 1, and so on.

#### 2.2.2. Definition of the Amino Acid K-Interval Product Factor (AAIPF(k))

In multiple protein monomers interactions, the individual behavior of the amino acid at each position is affected by the neighboring amino acids in the protein sequence. We define the amino acid k-interval product factor to describe the influence of neighboring amino acids on a given residue.

The AAIPF(k) is defined as follows: the numbers at two positions with interval k are multiplied and divided by k on the amino acid number sequence. The AAIPF(k) can be divided into: amino acid forward k-interval product factor (AAFIPF(k)) and amino acid backwards k-interval product factor (AABIPF(k)) (see Formulas (3)–(5)).

(3) $$\mathrm{AAIPF}(k)\;=\;\{\begin{array}{c} AAFIPF\left(k\right)\\ AABIPF\left(k\right) \end{array}$$

(4) $$\mathrm{AAFIPF}(k)\;=\;(\Phi_{j}^{i}*\Phi_{j-k}^{i})/k$$

(5) $$\mathrm{AABIPF}(k)\;=\;(\Phi_{j}^{i}*\Phi_{j+k}^{i})/k.$$

When exploring the individual behavior of each amino acid in a protein sequence P, as previously reported [27], we regard the protein sequence P as a cycle alphabet sequence with head-to-tail connections, and thus number sequences can also be regarded as cycle number sequences.

Considering the dimensionality of descriptors, and using the experience of previous works [28,29], we only used AAIPF(1), AAIPF(2), AAIPF(3), AAIPF(4), and AAIPF(5) to characterize each amino acid in the protein sequence P. In this way, each amino acid in the protein sequence P could be represented by the 10-dimensional characteristics of each numerical sequence. To reduce the redundancy between features, we only use the first five numerical sequences in formula 2 to calculate AAIPF(k). Thus, each amino acid in protein sequence P could be characterized by 50 features.

We also used as features the values of five physicochemical properties of amino acids, which we called basic first-order sequence features. Three versions of the hydrophobic values were used. Therefore, seven basic first-order sequence features were applied to describe an amino acid.

Considering the electrostatic interaction is also one of the important factors to stabilize the protein structure. Therefore, we used the electric property values (pK_1_ and pK_2_) as features to describe the residue. The values of pK_1_ and pK_2_ were calculated by the propka3.1 software [30].

#### 2.2.3. Residue Geometric Features

In several previous research studies [31,32,33], it has been found that accessible surface area (ASA) and relative solvent accessible surface area (RASA) play important roles in distinguishing between interface residues and noninterface residues. In addition to the above two geometric features, we also use three residue geometric features, exterior contact area (ECA), interior contact area (ICA), and exterior void area (EVA) extracted by our laboratory to describe each residue. These five geometric features were considered the basic structural geometric features. These five geometric features and their calculation tools are shown in Table 2.

ASA is the surface area of molecules that is accessible to solvents. Here, we used the Naccess V2.1.1 software [34] to calculate ASA. The RASA was used to describe the exposed or buried state of the residue and was calculated by formula 6. The specific definition of ECA, ICA, and EVA is given in detail elsewhere [26]. ECA and ICA were calculated by the Qcontacts software [35].

(6) $$RASA=\frac{ASA_{bound}}{ASA_{unbound}}$$

where $ASA_{bound}$ represents the solvent accessible surface area of the residue in the protein complex. $ASA_{unbound}$ is the solvent-accessible surface area of this residue in unbound state.

Through the above research and analysis, we extracted a total of 64 dimensional characteristics to describe each residue in the protein monomer. Therefore, we can use 128 dimensional characteristics to describe a residue pair formed by residues from two protein monomers. We used Formula (7) to standardize these 128 dimensional characteristics.

(7) $$x^{*}=\frac{x-min}{max-min}$$

### 2.3. Feature Vector Engineering

Different characteristics and their combinations may play different roles in the prediction of interactions interface residues pairs in multiple protein monomers. Therefore, we performed feature vector engineering using two sets of feature vectors to describe a residue pair. We used the 128 dimensional characteristics extracted as the first set of feature vectors. Considering that some of the 128 dimensional characteristics have a strong correlation, we deleted 1/4 of the characteristics, and used the remaining characteristics as the second set of feature vectors. We filtered the characteristics according to the Pearson correlation coefficients *r* and F-values. The specific process was as follows:

In the first step, 128 dimensional characteristics were clustered according to the Pearson correlation coefficient *r*. The formula of the Pearson correlation *r* is as follows:

(8) $$r=\frac{cov\left(X_{k},X_{l}\right)}{\sigma_{k}\sigma_{l}}$$

where $X_{k}$ and $X_{l}$ represent the k-th and l-th characteristics of the sample, respectively. $\sigma_{k}\;$ and $\sigma_{l}$ represent the mean square deviation of the k-th and l-th characteristics of the sample (sample: residue pairs), respectively.

In the second step, we used Formula (9) to calculate the F-value of each characteristic between the positive sample and the negative sample (negative sample: noninterface residue pairs and positive sample: interface residue pairs). The larger the F-value of the characteristic, the greater the difference between positive and negative samples and the greater the contribution of the characteristic to distinguish positive and negative samples.

(9) $$F(x_{m})=\left|\frac{\mu_{m}^{+}-\mu_{m}^{-}}{\sigma_{m}^{+}-\sigma_{m}^{+}}\right|$$

where $\mu_{m}^{+}$ and $\mu_{m}^{-}$ are the mean values of the m-th characteristic of the positive sample and the negative sample, respectively. $\sigma_{m}^{+}$ and $\sigma_{m}^{-}$ are the mean square deviation of the *m*-th characteristic of the positive sample and the negative sample, respectively.

We preserved basic first-order sequence characteristics and basic structural geometric characteristics. For the class with Pearson correlation coefficient |*r*| > 0.5, we preserved the characteristics of a relatively large F-value, with 96 characteristics in total. We used the 96 characteristics as the second set of feature vectors (see Appendix A Table A2).

### 2.4. Our Algorithms (A Two-Layer SVM Ensemble-Classifier)

Each protein trimer consists of three chains, and any two chains interact to form a protein–protein interaction interface. We split each protein trimer into three protein–protein interactions. Through Section 2.2 Feature Extraction and Section 2.3 Feature Vector Engineering, two sets of feature vectors were used to represent a residue pair. Therefore, we generated two sets of train data for the training trimer protein complexes. The train data composed of the first (second) set of feature vector was called Train data 1 (2).

The proportion of protein–protein interface residue pairs in all residue pairs was very low. Therefore, the positive and negative classes in train data 1 (2) were extremely imbalanced (negative classes: noninterface residue pairs and positive class: interface residue pairs). We used under-sampling to deal with the class imbalance problem. To improve the performance of the method, we used the classifier ensemble to alleviate the lack of information caused by the under-sampling. The process was as follows:

Generation of balanced subset samples (for training set j (j = 1,2)):

First, we randomly generated 100 subset samples of negative class from all the negative class samples in train data j. The total number of positive class was 12,687 in the entire training set. Therefore, we set the number of negative class in each subset sample to 12,687. Second, we combined each negative class subset sample with all positive class samples to generate a balanced subset sample. Finally, obtained 100 balanced subset samples for train data j (j = 1,2).

Generation of an ensemble classifier:

SVM is a supervised machine learning method, which is widely used in the field of protein–protein interactions. Here, we also used SVM to predict trimer protein complexes interface residue pairs.

**Figure 2:**
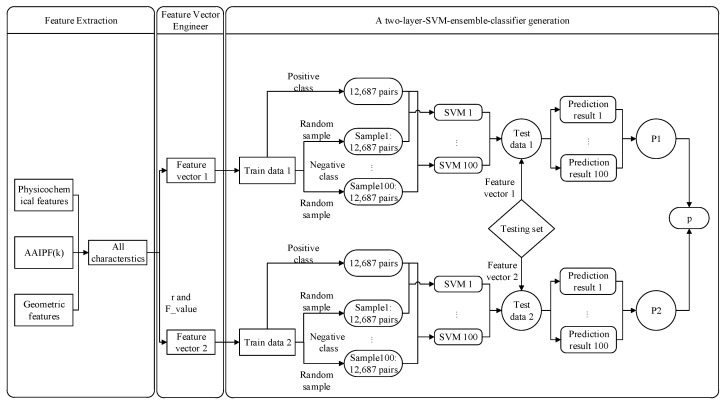
Flow chart of the two-layer support vector machine (SVM) ensemble-classifier method.

There are 100 balance subset samples in train data j (j = 1,2), each of which can be used to train an SVM model. We obtained 100 individual SVM predictors for train data j. We then developed an ensemble SVM classifier $P_{j}$ by fusing the 100 individual SVM predictors in train data j through a probability system, as shown in formula 10. Finally, a two-layer SVM ensemble-classifier P was formed by fusing two ensemble SVM classifiers P_1_ and P_2_ through a weight ω (here, we set ω to 1/2). We provide a flowchart in Figure 2 to illustrate how to generate a two-layer SVM ensemble-classifier P (An implementation of our model is available at the website ftp://202.112.126.135/pub/Trimer/code).

(10) $$P_{j}\left(x\right)=\sum_{i=1}^{100}SVM^{i}\left(x\right)\;(i\;=\;1,2,\text{…},100,\;j\;=\;1,2)$$

where $SVM^{i}$ indicates the SVM predictor trained with an i-balance subset sample. x indicates a residue pair. $SVM^{i}\left(x\right)$ indicates the probability that the residue pair x is the interface residue pair in the i-th individual SVM prediction of train data j.

(11) $$P\left(x\right)=\omega \times P_{1}\left(x\right)+\left(1-\omega \right)\times P_{2}\left(x\right)$$

### 2.5. Evaluation Criteria

The output of our model is a value between 0 and 1, showing the possibility of the residue pair to be an interface residue pair. The values were sorted from large to small. The number *t* predicted interface residue pairs with highest probability were used as the top t predicted interface residue pairs.

We used the following three measures to evaluate the performance of our method. First, we defined a three-dimensional vector $\;NPRPT\left(t\right)={\left(n_{1},n_{2},n_{3}\right)}_{t}$, where $n_{z}$ (z = 1,2,3) represents the number of positive interface residue pairs in the top t predictions for each possible protein–protein interface of a protein trimer. Here, NPRPT(t) is the abbreviation of the number of positive interface residue pairs in the top *t* predictions for each protein trimer. The first index is $\|NPRPT{\left(t)\right\|}_{0}$, which represents the L0 norm of NPRPT(t). It is consistent with the meaning of the L0 norm of vector in mathematics, which represents the number of nonzero elements in a vector. So $\|NPRPT{\left(t)\right\|}_{0}$ represents the number of interfaces that we can correctly predict in each protein trimer. In the top t predictions provided, if there is at least one positive interface residue pair, we assumed that the protein–protein interaction interface could be predicted correctly.

The second index is $\|NPRPT{\left(t)\right\|}_{1}$, which represents the L1 norm of NPRPT(t), see Formula (12). It is consistent with the meaning of the L1 norm of vector in mathematics. Therefore, $\|NPRPT{\left(t)\right\|}_{1}$ represents the number of positive interface residue pairs in the top t predictions at a protein trimer

(12) $$\|NPRPT{\left(t)\right\|}_{1}=n_{1}+n_{2}+n_{3}.$$

The third index is index accuracy rate (see Formula (13)).

(13) $$\mathrm{accuracy}\;\mathrm{rate}\;{\left(t\right)}_{z}=\frac{\mathrm{SCT}\left(t\right)}{TNT}\times 100\%.$$

SCT represents the sum of all correctly predicted trimer protein complexes. In the top *t* predictions, if there were z protein–protein interaction interfaces satisfying at least one positive interface residue pair, we assumed that the protein trimer was predicted correctly. TNT represents the total number of trimer protein complexes in the dataset.

## 3. Results and Discussion

### 3.1. Application of our Algorithms on the Testing Set

There were 26 trimer protein complexes in the testing set. Each protein trimer consists of three chains, and any two chains interact to form a protein–protein interaction interface. Therefore, we obtained 78 protein–protein interaction interfaces. Through Section 2.2 Feature Extraction and Section 2.3 Feature Vector Engineering, two sets of feature vectors can be used to represent a residue pair. Therefore, we can generate test data 1 and test data 2 for the testing trimer protein complexes. Then by inputting test data 1 and test data 2 to our proposed Algorithms, we can get the prediction results of the 78 protein–protein interaction interfaces.

### 3.2. Analysis of the Testing Set Results

Table 3 shows the top *t* (*t* = 10, 15, 20, and 30) predictions and the two evaluation indexes corresponding to the testing set results. It can be seen from Table 3 that when 3 protein–protein interaction interfaces of each protein trimer were correctly predicted, a total of 9 trimer protein complexes were correctly predicted in the top 10 predictions. The prediction result of 2IY0 protein trimer was the best, with up to 10 positive interface residue pairs. When 3 protein–protein interaction interfaces of each protein trimer are correctly predicted, a total of 17 trimer protein complexes were correctly predicted in the top 30 prediction results. Among them, there were 10 trimer protein complexes for which at least 10 positive interface residue pairs were predicted.

To further analyze the prediction results, we obtained the index $accuracy\;rate\;{\left(t\right)}_{z}$ from all $\|NPRP{T\|}_{0}$ columns in Table 3 (see Table 4). When 3 protein–protein interaction interfaces of each protein trimer were correctly predicted in the top 15 predictions, the accuracy rate was 42.31%, i.e., more than 2/5 of trimer protein complexes in the testing set were correctly predicted. When 3 protein–protein interaction interfaces of each protein trimer were correctly predicted in the top 30 predictions, the accuracy rate was as high as 65.38%. When at least 2 protein–protein interaction interfaces of each protein trimer are correctly predicted in the top 10 predictions, the accuracy rate was 53.85%, i.e., more than half trimer protein complexes in the testing set were correctly predicted. When at least 1 protein–protein interaction interface of each protein trimer was correctly predicted, the accuracy rate was 76.92% in the top 10 predictions and up to 92.31% in the top 30 predictions.

There are 6479 pairs of interface residue pairs in the test set, of which 968 pairs are formed by residues at N- and C-terminal regions, accounting for 15% (here, residues at the N- and C-terminal regions is the residues that we have specially treated in the manuscript). We can accurately predict 190 interface residue pairs for all testing set protein trimers, of which 48 pairs are formed by residues at N-terminal and C-terminal, accounting for 25%.

We compared the performance of our method with the previous method [16]. When at least 1 protein–protein interaction interface of each protein trimer was correctly predicted, the accuracy of our method is 76.92% and of the previous method [16] is 31.1% in the top 10 predictions. The accuracy of our method is higher than them.

**Figure 3:**
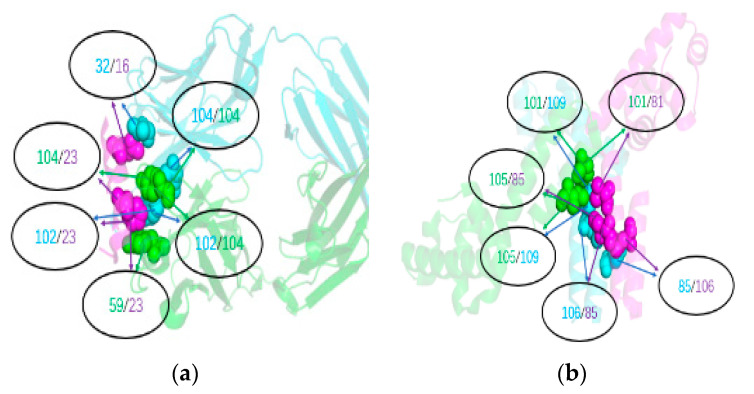
Experimental three-dimensional structure of the 3ffd and 1s7o trimer protein complexes. Figure (**a**) and Figure (**b**) are the three-dimensional structure of 3ffd and 1s7o protein trimer. We label three protein monomers with pink, blue, and green. The number of markers in the black circle indicates the correct predicted interface residue pair position on the two protein monomers.

The analysis of the above results showed that our proposed method was able to accurately predict the interface residue pairs of trimer protein complexes. Additionally, our predicted results are consistent with the experimental results. In the experimental article of the 3ffd protein trimer [36], it is mentioned that residues 20 and 24 are strictly conserved, which allows for extensive interactions with the antibody. Residues 16, 20, 27, 52, 59, 97, 102, and 104 are also binding sites. In our top 20 prediction results, we successfully predicted 8 positive interface residue pairs. For clarity, 6 positive interface residue pairs (Tyr 104-Phe 102, Tyr 104-Tyr 104, Tyr 104-Phe 23, Tyr 59-Phe 23, Phe 102-Phe 23, and Thr 32-Gln 16) for the 3ffd protein trimer are shown in Figure 3a. In the experimental article of the 1s7o protein trimer [37], it is pointed out that the 1s7o protein trimer has two structural domains and the primary interaction mainly involves the second central domain. The hydrophobic residues Ile 85, Phe 86, Met 89, Ile 90, Leu 99, Ile 103, and Leu 106 create both an intermolecular and intramolecular hydrophobic core in the second domain. Arg82 and Asp 110 form salt bridges, and two Arg82 guanidyl groups in adjacent molecules contribute to the intramolecular and intermolecular interactions. In our top 20 prediction results, we have successfully predicted 11 positive interface residues pairs formed by these residues and their surrounding residues. For illustration purposes, we show 6 positive interface residue pairs (Ile105-Ile109, Glu101-Ile 109, Ile 105-Ile 85, Glu 101-Val 81, Leu 106-Ile 85, and Ile 85-leu 106) in Figure 3b.

The training set contains a lot of antibody fragments, which make up two of the three chains: 1BGX, 3O2D, 3R1G. 3GI9, 1JPS, 1JRH, 1FNS. Similarly, 1F6F, 1EER, 1HWG, and 3VA2 are all cytokine receptor complexes with probable similarity between the receptor CRH domains. We deleted 1BGX, 3O2D, 3R1G. 3GI9, 1JPS, 1JRH, 1FNS 1F6F, 1EER, 1HWG, 3VA2 in the training set. The test set also contains 3 complexes with antibody chains: 3FFD, 1OSP, and 1SY6. We generated testing set 2, which deleted 3FFD, 1OSP, and 1SY6 relative to testing set. Appendix A Table A3 shows the top *t* (*t* = 15, 20, and 30) predictions and the two evaluation indexes corresponding to the testing set 2 results. We compared the prediction results of testing set with that of testing set 2 (Table 5). When at least 2 protein–protein interaction interfaces of each protein trimer are correctly predicted, the accuracy of testing set 2 is about 7% lower than that of testing set. When at least 3 protein–protein interaction interfaces of each protein trimer are correctly predicted in the top 30 predictions, the accuracy of testing set 2 is about 8.5% lower than that of testing set. When at least 3 protein–protein interaction interfaces of each protein trimer are correctly predicted in the top 20 predictions, the accuracy of testing set 2 was 6% higher than that of test set. The rest of the prediction results of the two test sets are almost the same.

### 3.3. Comparison with Random Results

We assume that the stochastic prediction of interface residue pairs of each protein–protein interaction interface in trimer protein complexes obeys a hypergeometric distribution X~H(N,M,T) [38]; where *X* is the number of positive interface residue pairs in the top *T* predictions. N is the number of all the residue pairs of one protein–protein interaction interface in one protein trimer. M is the number of positive interface residue pairs in this protein–protein interaction interface. Next, we can calculate the probability P that there are x positive interface residue pairs in the *T* predictions of one protein–protein interaction interface by the stochastic model (see Formula (14)):

(14) $$P\left(X=x\right)=\frac{C_{M}^{x}C_{N-M}^{T-x}}{C_{N}^{T}}.$$

In order to simplify the calculation, we assumed that each protein–protein interaction interface was independently identically distributed, and N is the mean value of all residue pairs in each protein–protein interaction interface, and M is the mean value of positive interface residue pairs in each protein–protein interaction interface. It can be seen that N is about 40,920 and M is about 83 in the Appendix A Table A4 When at least 1 protein–protein interaction interface of each protein trimer has at least one positive interface residue pair in *T* predictions, the probability $\tilde{P_{1}}$ is:

(15) $$\tilde{P_{1}}\left(X\geq 1\right)=1-\frac{C_{40,920-83}^{T}}{C_{40,920}^{T}}.$$

Consideration of the complexity of the $\tilde{P_{1}}$ calculation, we have made an enlarged calculation of $\tilde{P_{1}}$ (see inequality 16 and 17). Obviously, the computational complexity of the ${\hat{P}}_{1}$ is less than $\tilde{P_{1}}$, and when *T* is fixed, $\tilde{P_{1}}$ is less than ${\hat{P}}_{1}$. When the value of *T* is 10, 15, 20, and 30, we can calculate ${\hat{P}}_{1}$ through the Monte Carlo simulation method (see Table 6).

(16) $$\tilde{P_{1}}\left(X\geq 1\right)=1-\frac{C_{40,920-83}^{T}}{C_{40,920}^{T}}\leq 1-\frac{40,837-T+1}{40,920-T+1}$$

(17) $$\tilde{P_{1}}\leq {\hat{P}}_{1}\;({\hat{P}}_{1}=1-\frac{40,837-T+1}{40,920-T+1})$$

When at least 2 protein–protein interaction interfaces of each protein trimer have at least one positive interface residue pair in *T* predictions, the probability $\tilde{P_{2}}$ is:

(18) $$\tilde{P_{2}}=C_{3}^{2}{\tilde{P_{1}}}^{2}*\left(1-\tilde{P_{1}}\right)+{\tilde{P_{1}}}^{3}$$

Combining Formulas (15) and (18), we also enlarge $\tilde{P_{2}}$ and obtained inequality 20. Obviously, the computational complexity of the ${\hat{P}}_{2}$ is much less than $\tilde{P_{2}}$, and when *T* is fixed, $\tilde{P_{2}}$ is less than ${\hat{P}}_{2}$. When the value of *T* is 10, 15, 20, and 30, we can calculate ${\hat{P}}_{2}$ through the Monte Carlo simulation method (see Table 6).

(19) $$\begin{array}{c} \tilde{P_{2}}=C_{3}^{2}{\tilde{P_{1}}}^{2}\times \left(1-\tilde{P_{1}}\right)+{\tilde{P_{1}}}^{3}\\ =1-3{\left(\frac{40,837\times 40,836\times \cdots \times \left(40,837-T+1\right)}{40,920\times 40,919\times \cdots \times \left(40,920-T+1\right)}\right)}^{2}+2{\left(\frac{40,837\times 40,836\times \cdots \times \left(40,837-T+1\right)}{40,920\times 40,919\times \cdots \times \left(40,920-T+1\right)}\right)}^{3}\\ \leq 1-3{\left(\frac{40,837-T+1}{40,920-T+1}\right)}^{2}+2{\left(\frac{40,837}{40,920}\right)}^{3} \end{array}$$

(20) $$\tilde{P_{2}}\leq {\hat{P}}_{2}({\hat{P}}_{2}=1-3{\left(\frac{40,837-T+1}{40,920-T+1}\right)}^{2}+2{\left(\frac{40,837}{40,920}\right)}^{3})$$

When 3 protein–protein interaction interfaces of each protein trimer have at least one positive interface residue pair in *T* predictions, the probability $\tilde{P_{3}}$ is:

(21) $$\tilde{P_{3}}={\tilde{P_{1}}}^{3}$$

(22) $$\tilde{P_{3}}={\tilde{P_{1}}}^{3}={\left(1-\frac{40,837\times 40,837\times \cdots \times \left(40,837-T+1\right)}{40,920\times 40,919\times \cdots \times \left(40,920-T+1\right)}\right)}^{3}\leq {\left(1-\frac{40,837-T+1}{40,920-T+1}\right)}^{3}$$

(23) $$\tilde{P_{3}}\leq {\hat{P}}_{3}\;\left({\hat{P}}_{3}={\left(1-\frac{40,837-T+1}{40,920-T+1}\right)}^{3}\right)$$

In the same way as above, we also enlarged $\tilde{P_{3}}$ and obtained ${\hat{P}}_{3}$ (see Inequality (22) and (23)). When the value of *T* was 10, 15, 20, and 30, we calculated ${\hat{P}}_{3}$ through the Monte Carlo simulation method (see Table 6).

As can be seen from Table 5, the accuracy of our method to predict the interface residue pairs of trimer protein complexes is much higher than that of random results. When at least 1 protein–protein interaction interface of each protein trimer was correctly predicted, our method accuracy was over 76.92% and up to 92.31%, while the random accuracy was lower than 0.20298%. When at least 2 protein–protein interaction interfaces of each protein trimer were correctly predicted, our accuracy was over 53.85% and up to 84.62%, whereas the random accuracy was below 0.0015%. When 3 protein–protein interaction interfaces of each protein trimer were correctly predicted, our accuracy achieved 65.38% in the top 30 predictions, and the accuracy was more than 108 times higher than that of random results.

## 4. Conclusions

In this paper, we defined an amino acid k-interval product factor to describe the influence of neighboring amino acids on a residue. This method takes advantage of the physicochemical and geometric properties of amino acids, and also considers the influence of neighboring amino acids (amino acid k-interval product factor) as features. Finally, we developed a two-layer SVM ensemble-classifier method, based on feature vector engineering and SVM, to predict the interface residue pairs of trimer protein complexes. In our testing set, the accuracy rate of successfully predicting one interface was 84.62%, and for two interfaces, the accuracy rate was 73.08%, in the top 15 predictions, which indicates significance for biological experimentation and biomedical-related research. Moreover, our predicted results are consistent with the experimental results. This shows that our method is effective and reliable to predict interface residue pairs of trimer protein complexes. However, our accuracy rate was not high when three interfaces of one trimer are required to predict correctly. We also did not consider protein conformational changes. These are the areas where we will improve in the future.

## Figures and Tables

**Table 1:** Detailed information of the training set and testing set.

Data Set Name	PDB Code												
Training Set	1A12	1AHS	1AWI	1B77	1BGX	1CJD	1CUN	1DKG	1EER	1EL6	1F6F	1FNS	1FPO
	1G2X	1HWG	1IDP	1IK9	1J5S	1JPS	1JRH	1KI9	1KKE	1L5A	1LW1	2ADV	2AZE
	2B2Y	2B4I	2BSD	2CU5	2DJ6	2E2A	2E4M	2FB5	2FM8	2FVH	2FZ1	2GDG	2GMI
	2I15	2P90	2PBQ	3CC0	3EMF	3F5C	3G65	3GI9	3N4G	3NAP	3O2D	3R1G	3VA2
Testing Set	1OSP	1OY3	1P32	1Q5X	1QB3	1S7O	1SG2	1STZ	1SY6	1W9Z	1WDJ	1YNB	1ZA7
	2IG8	2IUM	2IY0	2IZW	2MS2	2R3U	2WR5	3DLI	3FFD	3M6N	3OWT	3P5J	3QKS

**Table 2:** The five geometric features and their calculation tools.

Features	Abbreviation	Software or Researchers
Accessible surface area	ASA	Naccess V2.1.1
Relative accessible surface area	RASA	Naccess V2.1.1
Exterior contact area	ECA	Qcontacts
Interior contact area	ICA	Qcontacts
Exterior void area	EVA	NACCES V2.1.1, Qcontacts

**Table 3:** Two evaluation indexes of the testing set prediction results.

**Protein Name**	t=10		t=15		t=20		t=30	
	$\|NPRP{T\|}_{0}$	$\|NPRP{T\|}_{1}$	$\|NPRP{T\|}_{0}$	$\|NPRP{T\|}_{1}$	$\|NPRP{T\|}_{0}$	$\|NPRP{T\|}_{1}$	$\|NPRP{T\|}_{0}$	$\|NPRP{T\|}_{1}$
1osp	2	3	2	3	2	5	3	10
1oy3	3	4	3	5	3	6	3	9
1p32	1	3	1	3	1	3	1	4
1q5x	3	5	3	5	3	6	3	10
1qb3	3	4	3	4	3	4	3	9
1s7o	3	7	3	9	3	11	3	14
1sg2	2	3	3	5	3	7	3	9
1stz	0	0	0	0	0	0	0	0
1sy6	2	2	2	2	2	3	2	5
1w9z	3	3	3	3	3	3	3	7
1wdj	2	2	2	4	2	6	2	8
1ynb	2	2	2	3	2	4	2	4
1za7	1	4	2	6	3	9	3	12
2ig8	1	1	1	2	2	3	3	5
2ium	1	2	3	4	3	6	3	9
2iy0	3	10	3	14	3	16	3	19
2izw	1	2	2	3	2	4	3	9
2ms2	3	5	3	8	3	8	3	10
2r3u	0	0	0	0	1	1	2	2
2wr5	0	0	0	0	0	0	0	0
3dli	3	3	3	3	3	3	3	6
3ffd	3	4	3	4	3	8	3	10
3m6n	0	0	0	0	0	0	1	1
3owt	1	3	2	4	2	5	2	8
3p5j	0	0	2	2	2	3	3	4
3qks	0	0	1	1	2	2	3	6

**Table 4:** $\mathrm{Accuracy}\;\mathrm{rate}\;{\left(t\right)}_{z}$ of the testing set prediction results

	t	t=10	t=15	t=20	t=30
z					
z=3		34.62%	42.31%	46.15%	65.38%
z=2		53.85%	73.08%	80.77%	84.62%
z=1		76.92%	84.62%	88.46%	92.31%

**Table 5:** $\mathrm{Accuracy}\;\mathrm{rate}\;{\left(t\right)}_{z}$ of the testing set and testing set 2 prediction results.

	t	t=15		t=20		t=30	
z		Result1	Result2	Result1	Result2	Result1	Result2
z=3		42.31%	43.48%	46.15%	52.17%	65.38%	56.52%
z=2		73.08%	65.22%	80.77%	73.91%	84.62%	78.26%
z=1		84.62%	82.61%	88.46%	86.96%	92.31%	91.30%

a. Result1 represents the testing set result accuracy. b. Result2 represents the accuracy of the result in the testing set 2.

**Table 6:** Comparison of our method with that of random results.

Accuracy Rate	t=10	t=15	t=20	t=30
${\hat{P}}_{1}$	0.20288%	0.20290%	0.20292%	0.20298%
$\mathrm{accuracy}\;\mathrm{rate}\left(t\right){\;}_{1}$	76.92%	84.62%	88.46%	92.31%
${\hat{P}}_{2}$	0.001500%	0.001648%	0.001797%	0.00002094%
$\mathrm{accuracy}\;\mathrm{rate}\left(t\right){\;}_{2}$	53.85%	73.08%	80.77%	84.62%
${\hat{P}}_{3}$	0.0000008351%	0.0000008354%	0.0000008357%	0.0000008363%
$\mathrm{accuracy}\;\mathrm{rate}\left(t\right){\;}_{2}$	34.62%	42.31%	46.15%	65.38%

## Supplementary Materials

Code: ftp://202.112.126.135/pub/Trimer/code. Data: ftp://202.112.126.135/pub/Trimer.

## Appendix A

**Table A1:** Five physicochemical properties for the 20 amino acids.

Amino Acid	Φ^1^	Φ^2^	Φ^3^	Φ^4^	Φ^5^	Φ^6^	Φ^7^
A	0.62	0.046	8.1	−1.302	1.57	0.17	0.50
C	0.29	0.128	5.5	0.465	−1.02	−0.24	−0.02
D	−0.9	0.105	13	0.302	−0.259	1.23	3.64
E	−0.74	0.151	12.3	−1.453	0.113	2.02	3.63
F	1.19	0.29	5.2	−0.59	−0.397	−1.13	−1.71
G	0.48	0	9	1.652	1.045	0.01	1.15
H	−0.4	0.23	10.4	−0.417	−1.474	0.96	2.33
I	1.38	0.186	5.2	−0.547	0.393	−0.31	−1.12
K	−1.5	0.219	11.3	−0.561	−0.277	0.99	2.80
L	1.06	0.186	4.9	−0.987	1.266	−0.56	−1.25
M	0.64	0.221	5.7	−1.524	−1.005	−0.23	−0.67
N	−0.78	0.134	11.6	0.828	−0.169	0.42	0.85
P	0.12	0.131	8	2.081	0.421	0.45	0.14
Q	−0.85	0.18	10.5	−0.179	−0.503	0.58	0.77
R	−2.53	0.291	10.5	−0.055	0.44	0.81	1.81
S	−0.18	0.062	9.2	1.399	0.67	0.13	0.46
T	−0.05	0.108	8	0.326	0.908	0.14	0.25
V	1.08	0.14	5.9	−0.279	1.242	0.07	−0.46
W	0.81	0.409	5.4	0.009	−2.128	−1.85	−2.09
Y	0.26	0.298	6.2	0.83	−0.838	−0.94	−0.71

We used Φ^1^, Φ^2^, Φ^3^, Φ^4^, Φ^5^, Φ^6^, and Φ^7^ to represent the hydrophobicity 1, polarizability, polarity, secondary structure, codon diversity, hydrophobicity 2, and hydrophobicity 3 for the 20 amino acids.

**Table A2:** Forty-eight characteristics to describe a residue.

A Residue of Protein Monomer Characteristics					
ASA_A	Φ4	AAFIPF^1^(5)	AABIPF^2^(4)	AAFIPF^4^(5)	AAFIPF^5^(3)
RASA_A	Φ5	AABIPF^1^(1)	AABIPF^3^(1)	AABIPF^4^(1)	AAFIPF^5^(4)
ECA_A	Φ6	AABIPF^1^(2)	AABIPF^3^(2)	AABIPF^4^(2)	AAFIPF^5^(5)
ICA_A	Φ7	AABIPF^1^(3)	AABIPF^3^(5)	AABIPF^4^(3)	AABIPF^5^(1)
EVA_A	AAFIPF^1^(1)	AABIPF^1^(4)	AAFIPF^4^(1)	AABIPF^4^(4)	AABIPF^5^(2)
Φ1	AAFIPF^1^(2)	AABIPF^1^(5)	AAFIPF^4^(2)	AABIPF^4^(5)	AABIPF^5^(3)
Φ2	AAFIPF^1^(3)	AAFIPF^2^(2)	AAFIPF^4^(3)	AAFIPF^5^(1)	AABIPF^5^(4)
Φ3	AAFIPF^1^(4)	AABIPF^2^(2)	AAFIPF^4^(4)	AAFIPF^5^(2)	AABIPF^5^(5)

We used Φ1, Φ2, Φ3, Φ4, Φ5, Φ6, and Φ7 to represent basic first-order sequence features.We use 48 characteristics to describe a residue in the second set of feature vector.

**Table A3:** Two evaluation indexes of the testing set 2 prediction results.

Protein Name	t=15		t=20		t=30	
	$\|NPRP{T\|}_{0}$	$\|NPRP{T\|}_{1}$	$\|NPRP{T\|}_{0}$	$\|NPRP{T\|}_{1}$	$\|NPRP{T\|}_{0}$	$\|NPRP{T\|}_{1}$
1osp	3	5	3	6	3	10
1oy3	1	3	1	4	1	5
1p32	3	6	3	6	3	10
1q5x	3	4	3	5	3	9
1qb3	3	11	3	13	3	17
1s7o	3	7	3	9	3	10
1sg2	1	1	1	1	1	1
1stz	2	2	3	3	3	5
1sy6	2	4	2	5	2	7
1w9z	1	2	2	3	2	4
1wdj	3	8	3	11	3	12
1ynb	1	1	1	2	2	4
1za7	3	6	3	6	3	8
2ig8	3	12	3	14	3	21
2ium	2	3	2	4	3	9
2iy0	3	6	3	8	3	9
2izw	0	0	0	0	1	1
2ms2	0	0	0	0	0	0
2r3u	3	3	3	4	3	7
2wr5	0	0	0	0	0	0
3dli	2	5	2	6	2	7
3ffd	0	0	2	2	2	3
3m6n	2	3	3	5	3	8

**Table A4:** All residue pairs and positive interface residue pairs in each protein–protein interface.

Protein–Protein Interface	Positive Residue Pairs	All Residue Pairs
1osp_H_L	160	46,652
1osp_H_O	30	54,718
1osp_L_O	26	53,714
1oy3_B_C	53	15,120
1oy3_B_D	38	24,640
1oy3_C_D	86	29,700
1p32_A_B	93	31,122
1p32_A_C	95	32,032
1p32_B_C	94	30,096
1q5x_A_B	42	25,238
1q5x_A_C	37	24,800
1q5x_B_C	41	24,490
1qb3_A_B	7	13,447
1qb3_A_C	172	12,317
1qb3_B_C	15	12,971
1s7o_A_B	12	11,130
1s7o_A_C	21	11,448
1s7o_B_C	78	11,340
1sg2_A_B	44	15,369
1sg2_A_C	72	20,022
1sg2_B_C	50	15,478
1stz_A_B	53	100,453
1stz_A_C	135	100,453
1stz_B_C	45	96,721
1sy6_A_H	37	36,792
1sy6_A_L	15	35,784
1sy6_H_L	164	46,647
1w9z_A_B	150	66,049
1w9z_A_C	149	65,278
1w9z_B_C	152	65,278
1wdj_A_B	153	28,272
1wdj_A_C	45	34,596
1wdj_B_C	9	28,272
1ynb_A_B	197	27,889
1ynb_A_C	25	27,889
1ynb_B_C	84	27,889
1za7_A_B	43	24,915
1za7_A_C	31	24,915
1za7_B_C	33	27,225
2ig8_A_B	98	20,306
2ig8_A_C	104	20,164
2ig8_B_C	102	20,306
2ium_A_B	86	44,521
2ium_A_C	88	44,521
2ium_B_C	88	44,521
2iy0_A_B	90	17,176
2iy0_A_C	28	35,256
2iy0_B_C	13	11,856
2izw_A_B	88	31,862
2izw_A_C	80	37,024
2izw_B_C	90	37,232
2ms2_A_B	39	16,641
2ms2_A_C	36	16,641
2ms2_B_C	41	16,641
2r3u_A_B	88	39,390
2r3u_A_C	84	37,370
2r3u_B_C	97	36,075
2wr5_A_B	169	235,225
2wr5_A_C	176	235,225
2wr5_B_C	162	235,225
3dli_A_B	86	48,841
3dli_A_C	86	48,841
3dli_B_C	84	48,841
3ffd_A_B	150	45,570
3ffd_A_P	40	3780
3ffd_B_P	26	3906
3m6n_A_B	425	70,752
3m6n_A_C	68	69,696
3m6n_B_C	74	70,752
3owt_A_B	8	21,904
3owt_A_C	59	2960
3owt_B_C	31	2960
3p5j_A_B	67	44,802
3p5j_A_C	108	30,392
3p5j_B_C	214	19,836
3qks_A_B	267	35,621
3qks_A_C	29	4179
3qks_B_C	24	3759
mean	83.0641	40,919.63

Protein–protein interface column denotes the protein–protein interface of those two chains in a protein trimer, such as 1osp_ H_ L is the interaction interface between H chain and L chain of 1osp protein trimer.
